# Comparison of ultrasonography-guided fine-needle aspiration and endoscopic ultrasonography-guided fine needle aspiration for the diagnosis of pancreatic malignancy

**DOI:** 10.1016/j.clinsp.2026.101084

**Published:** 2026-09-05

**Authors:** Shiming Jiang, Yachao Zhu, Panguo Wang, Zhaowei Wu, Yong Chen

**Affiliations:** Department of Hepatobiliary Surgery, The First Affiliated Hospital of Chongqing Medical University, Yuzhong District, Chongqing, China

**Keywords:** Ultrasound fine needle aspiration, Endoscopic ultrasound fine needle aspiration, Pancreatic cancer

## Abstract

**Objective:**

Both Ultrasonography-guided Fine Needle Aspiration (US-FNA) and Endoscopic Ultrasonography-guided Fine Needle Aspiration (EUS-FNA) are techniques commonly used for diagnosing pancreatic malignancies. However, comparative studies evaluating the diagnostic effectiveness of different imaging modalities in detecting pancreatic cancer are lacking.

**Method:**

A retrospective study of patients diagnosed with unresectable pancreatic cancer who underwent at least one imaging technique and underwent US-FNA or EUS-FNA between January 2019 and October 2022 was conducted. All patients’ medical records were reviewed, and the following clinical data were extracted: sex, age, location and size of the lesion, uncinate, hypoechoic appearance, adequate specimen collection, time of pathological diagnosis, severe complications, repeat biopsy and pathology. To mitigate this bias, we implemented Propensity Score Matching (PSM). After matching, 35-pairs of patients were included.

**Results:**

Among these patients, there was no significant difference in age, sex, location or size of the lesion, repeat biopsy or complications between US-FNA and EUS-FNA. Adequate specimens were obtained from 35 of 35 US-FNA patients and 29 of 35 EUS-FNA patients (100%vs. 82.9%). The rate of inconclusive pathological diagnosis was lower among patients who underwent US-FNA than those who underwent EUS-FNA (5.71%vs. 8.57%). US-FNA demonstrated a diagnostic accuracy of 82.9%, whereas EUS-FNA also had a diagnostic accuracy of 82.9%.

**Conclusion:**

US-FNA is an effective and safe option compared with EUS-FNA in the diagnosis of pancreatic malignancy. It is good choice to establish a definitive diagnosis with key biomarkers.

## Highlights

• Ultrasonography-guided fine-needle aspiration achieves a higher specimen acquisition rate.
• Ultrasonography-guided and endoscopic ultrasonography-guided fine-needle aspiration have similar diagnostic accuracy for pancreatic malignancy.
• No significant differences are observed in race, lesion size, location between groups, repeat biopsy rates and complications.

## Introduction

Pancreatic cancer is a highly deadly cancer with a poor prognosis. It is the seventh most common cause of cancer-related death worldwide for both males and females.^1^ While surgical resection is generally viewed as the primary treatment for pancreatic cancer, unfortunately, the majority of patients with pancreatic cancer present with locally advanced or metastatic disease and are considered unresectable.^2^ Indeed, pathological diagnosis is essential in selecting a suitable treatment approach for patients with pancreatic cancer. Image-guided biopsies, specifically Endoscopic Ultrasonography (EUS)-guided Fine Needle Aspiration (FNA) and Ultrasonography-guided Fine Needle Aspiration (US-FNA), are valuable tools for diagnosing pancreatic tumours.^3^^,^^4^ Nevertheless, there is evidence indicating that EUS-FNA samples collected from pancreatic lesions may occasionally be inadequate for precise diagnosis.^5^ In such situations, alternative biopsy techniques, such as US-FNA, may be beneficial for overcoming this limitation and obtaining more satisfactory specimens.^6^ US-FNA is an image-guided biopsy technique that uses ultrasonographic imaging to guide the needle into the target lesion. Notably, US-FNA has demonstrated a higher diagnostic accuracy in patients with pancreatic tumours compared with EUS-FNA and offers several benefits for obtaining more satisfactory specimens. One of these advantages is the ability to employ diverse imaging modalities, such as transabdominal ultrasonography or endorectal ultrasonography, dictated by the location and accessibility of the pancreatic lesion. This study was conducted to compare the effectiveness and safety of EUS-FNA and US-FNA for diagnosing pancreatic malignancy. This study aimed to evaluate the diagnostic yield of these two biopsy techniques and assess their respective advantages and limitations.

## Methods

### Patient population

This analysis was conducted at Chongqing Medical University, and radiological and pathological data were obtained from 137 patients with pancreatic cancer. This retrospective study was approved by IRB. The study took place between January 2019 and October 2022, and all participants provided written informed consent. The patients were divided into two groups. Group A comprised 44 patients who underwent US-FNA, and Group B comprised 93 patients who underwent EUS-FNA. The procedures were performed by the same operator. The inclusion criteria for this study included the following: 1) Patients with a pancreatic malignancy accessible by at least one imaging modality, 2) Patients with a platelet count higher than 50×10^9/L or an International Normalized Ratio (INR) <2, and 3) Patients with history of prior chemotherapy or radiation therapy for pancreatic malignancy. Patients with acute pancreatitis or recurring chronic pancreatitis were excluded. Studies of diagnostic accuracy has been implemented in this manuscript. The study follows the STROBE Statement.

**Fig. 1:**
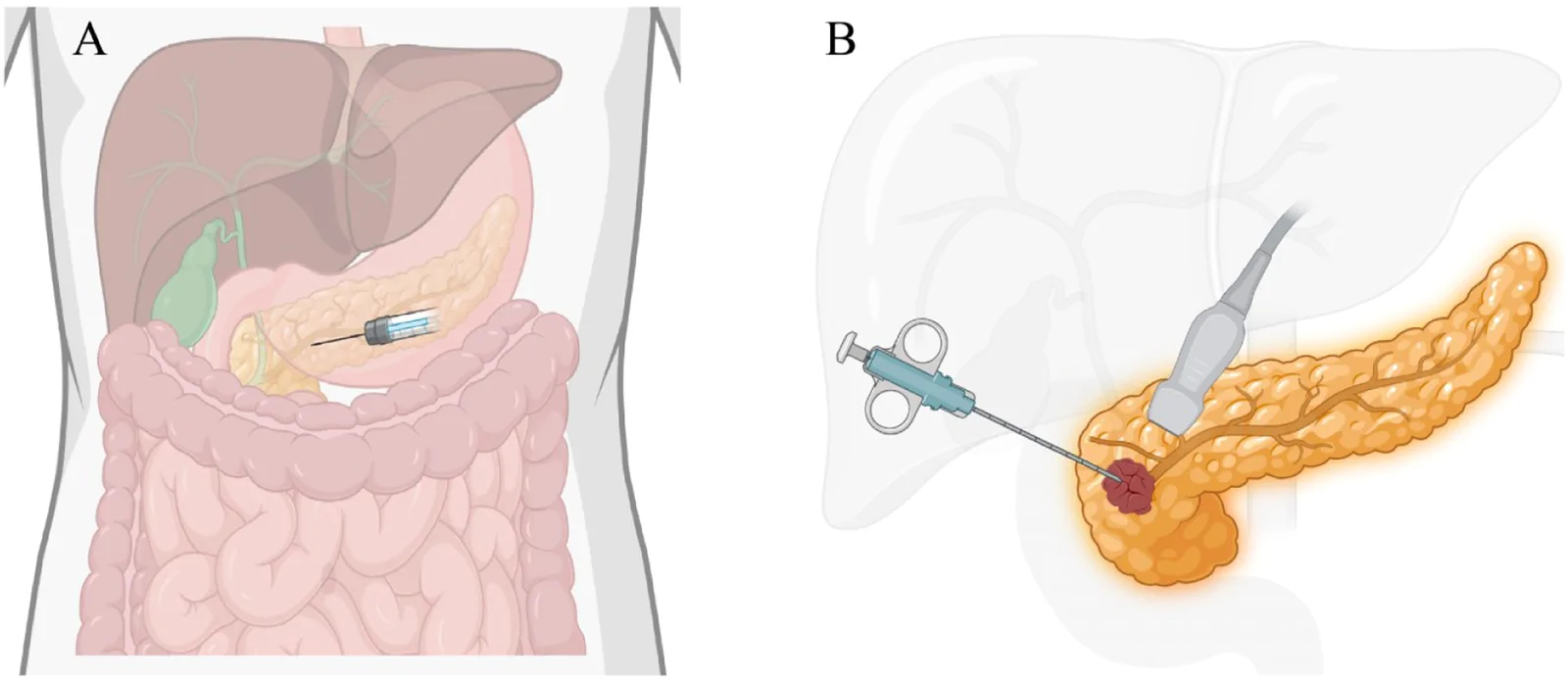
US-FNA Procedure. (A) Skin disinfection and local anesthesia with 1% lidocaine. (B) Ultrasound-guided lesion localization and puncture.

US-FNA were performed with the Aplio 1800 machine, which is a diagnostic ultrasound system manufactured by Toshiba Medical Systems Corporation (now Canon Medical Systems Corporation), Japan. This imaging technique is employed to visualize the pancreas and identify any abnormalities or lesions. Before the biopsy, the skin and transabdominal needle tract were sterilized, most likely using an antiseptic solution. To minimize pain, 1% lidocaine, a local anaesthetic, was likely administered at the puncture site. During the biopsy procedure, an ultrasonographic examination was performed to locate and target the pancreatic lesion accurately with a cutting needle (VPA1816). Ultrasonographic imaging was used to guide the placement of the needle for sampling the tissue of the identified lesion. The FNA technique was employed to obtain tissue specimens from the targeted pancreatic lesion. The cutting needle was likely used to collect the samples during the procedure. These specimens were then fixed in 10% formalin, a common fixative solution used to preserve tissue for subsequent pathological analysis. The procedures were showed in Fig. 1.

**Fig. 2:**
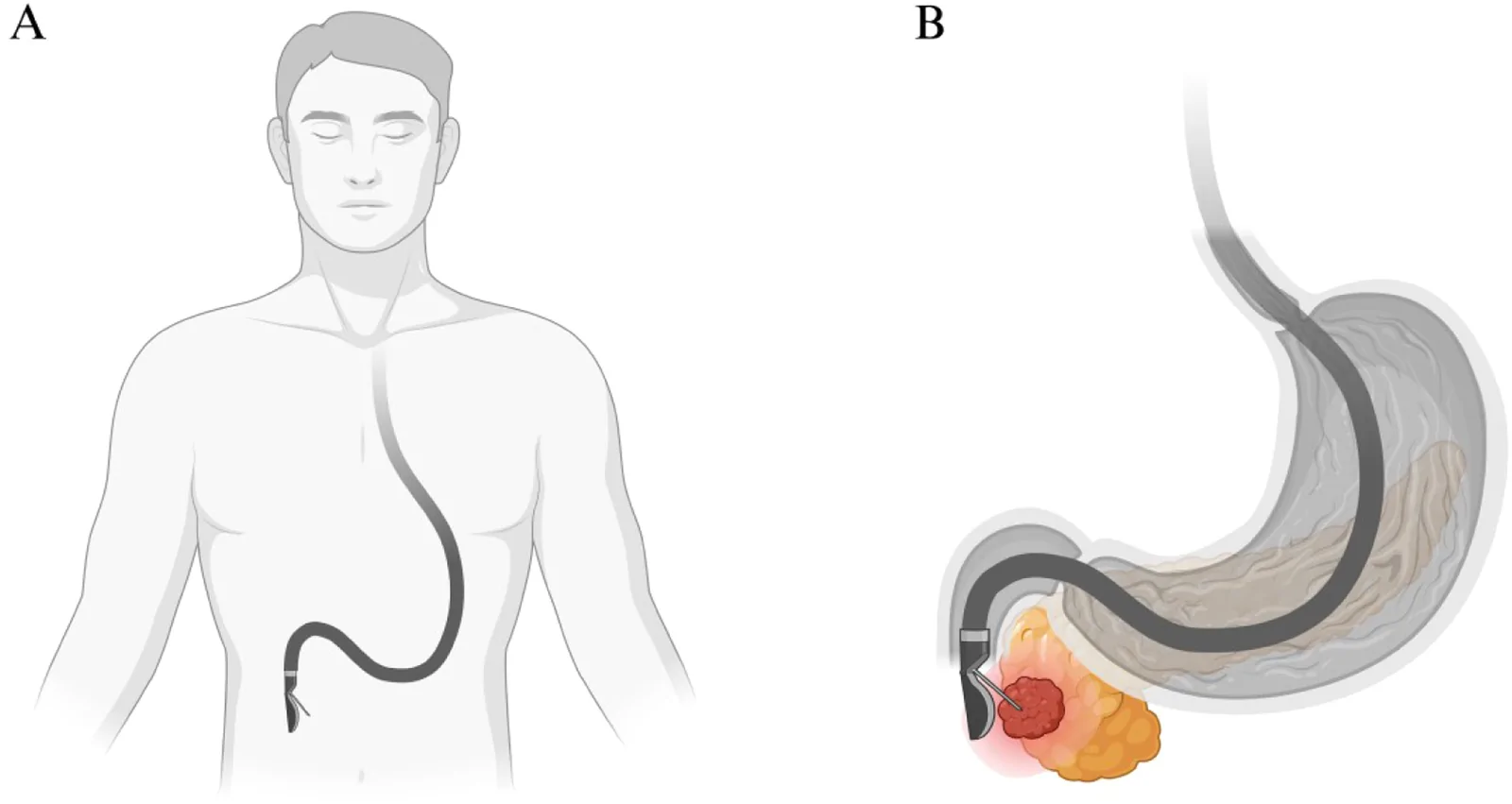
EUS-FNA Procedure. (A) Endoscopic ultrasound exploration under general anesthesia. (B) Targeted lesion sampling via multiple needle passes.

EUS-FNA procedures were performed with an echoendoscope, specifically the Olympus SV-260 model from Japan. This echoendoscope is designed for endoscopic ultrasonographic examinations and interventions and promotes detailed visualization of the common bile duct and pancreas. During the EUS-FNA procedures, a 22-gauge needle manufactured by Olympus (Japan) was utilized. This needle is specifically designed for fine-needle aspiration and sampling of tissues under endoscopic ultrasonographic guidance. Prior to the FNA, an initial echoendoscopic examination was performed to assess the common bile duct and pancreas. This step likely involved manoeuvring the echoendoscope through the gastrointestinal tract to obtain high-resolution ultrasonographic images of the target area for localization of the pancreatic lesion. Once the pancreatic lesion was localized, multiple aspiration punctures were performed to ensure that sufficient and representative tissue specimens were obtained. This step aimed to maximize the chances of acquiring an adequate sample for subsequent analysis. The obtained tissue specimens were fixed in 10% formalin, which is a standard fixative solution for histological preservation. Fixation in formalin helps preserve the cellular structure and prevents tissue degradation, allowing for subsequent histological examination. The fixed specimens were likely sent to the pathology laboratory for further processing. The specimens were processed for histological analysis, where thin sections of the tissue were prepared, stained, and examined under a microscope. Additionally, fragments of the tissue were taken for cytological analysis, which involves examining individual cells for abnormalities. The procedures were showed in Fig. 2.

A successful biopsy requires both technical success ‒ obtaining an adequate tissue sample ‒ and clinical success, where the pathology correlates with other findings. A pathological diagnosis of a tumor lesion is definitive, even without support from tumor markers, and is the final arbiter. In contrast, an indeterminate pathology result cannot fully rule out cancer, as some such cases are later confirmed during follow-up, potentially necessitating a repeat procedure for diagnosis. Moreover, complete blood count tests and serum amylase levels were obtained. Through consideration of the findings from imaging, cytology, histology, and clinical follow-up, the medical team would have arrived at a final diagnosis of the pancreatic tumour.

### Statistical analysis

The data were presented as frequencies and percentages. To mitigate this bias, PSM was implemented using logistic regression to estimate propensity scores, incorporate key covariates such as age, gender, lesion size, and lesion location. 1:1 nearest-neighbor matching without replacement and a caliper of 0.2 are performed. To assess the differences between the EUS-FNA and US-FNA groups, two statistical tests, the Survey-weighted logistic regression and Fisher’s exact test, were employed. A p-value threshold of <0.05 was considered statistically significant. The statistical analyses were performed using *R*, specifically version 4.5.0.

## Results

A total of 137 patients participated in this study; after PSM, 35 pairs of patients were included (70 total). 35 patients underwent US-FNA (Group A) and 35 patients underwent EUS-FNA (Group B). Before matching, significant differences were observed between the two groups in lesion size (SMD = 0.724), lesion location (e.g., pancreatic body, SMD = −0.569). After matching, all 35 matched pairs exhibited Standardized Mean Differences (SMDs) below 0.3 for every covariate. Specifically, SMDs for lesion location (head, tail) and lesion size approached 0, indicating well-balanced baseline characteristics between the groups (Table 1). The patients’ characteristics after PSM are shown in Table 2. The biopsy pathology results revealed various diagnoses, including Pancreatic Adenocarcinoma (PAAD), serous cystadenoma of the pancreas, mucinous carcinoma of the pancreas, pancreatic Neuroendocrine Tumours (NETs), inconclusive results (dysplasia) and no cancer. The histological results revealed that cancer tissue was present in 28 of the 35 patients (80%) in Group A and in 27 of the 35 patients (77%) in Group B. These findings indicate that most of the patients in both groups had cancerous tissue confirmed by histological analysis. In Group A, 26 patients were diagnosed with PAAD. A patient was diagnosed with mucinous cystadenoma and one patient with a NETs. In Group B, 26 patients were diagnosed with Pancreatic Adenocarcinoma (PAAD) and a patient was diagnosed with mucinous cystadenomas.

**Table 1:** Patient characteristics before and after propensity score matching.

	Before PSM		After PSM		Standardized difference	
Characteristic	EUS-FNA (n = 93)	US-FNA (n = 44)	EUS-FNA (n = 35)	US-FNA (n = 35)	Before PSM	After PSM
Age, years	60.4 ± 12.3	63.7 ± 10.8	62.9 ± 11.5	62.3 ± 10.2	0.348	−0.063
Gender					0.198	0.232
Male	64 (68.8%)	26 (59.1%)	18 (51.4%)	22 (62.9%)		
Female	29 (31.2%)	18 (40.9%)	17 (48.6%)	13 (37.1%)		
Tumor Location						
Head	40 (43.0%)	22 (50.0%)	20 (57.1%)	20 (57.1%)	0.140	0.000
Body	10 (10.8%)	1 (2.3%)	2 (5.7%)	1 (2.9%)	−0.569	−0.192
Neck	24 (25.8%)	5 (11.4%)	3 (8.6%)	4 (11.4%)	−0.455	0.090
Tail	19 (20.4%)	16 (36.4%)	10 (28.6%)	10 (28.6%)	0.331	0.000
Lesion Size, cm	3.1 ± 1.8	4.5 ± 2.1	3.8 ± 1.6	3.9 ± 1.9	0.724	0.096
Propensity Score	0.25 ± 0.15	0.48 ± 0.18	0.39 ± 0.14	0.40 ± 0.16	0.981	0.053

Data are presented as mean ± standard deviation or n (%). PSM, Propensity Score Matching. Standardized differences < 0.1 indicate adequate balance between groups.

**Table 2:** Patient characteristics after propensity score matching.

Characteristic		EUS-FNA (n = 35)	US-FNA (n = 35)
Age		62.9 ± 11.3	62.3 ± 9.4
Gender	F	17 (48.6%)	13 (37.1%)
	M	18 (51.4%)	22 (62.9%)
Location	Body	2 (5.7%)	1 (2.9%)
	Head	20 (57.1%)	20 (57.1%)
	Neck	3 (8.6%)	4 (11.4%)
	Tail	10 (28.6%)	10 (28.6%)
Lesion_size		3.8 ± 1.2	3.9 ± 1.1
Adequate_speciments_obtained	No	6 (17.1%)	0 (0%)
	Yes	29 (82.9%)	35 (100%)
Biopsy_time		2.5 ± 1.5	4.1 ± 3
Complication	No	34 (97.1%)	33 (94.3%)
	Fever	0 (0%)	1 (2.9%)
	Pancreatitis	1 (2.9%)	0 (0%)
	Intra-abdominal infection	0 (0%)	1 (2.9%)
Repeat_biopsy	No	34 (97.1%)	31 (88.6%)
	Yes	1 (2.9%)	4 (11.4%)
Pathology	No Cancer	5 (14.3%)	5 (14.3%)
	PAAD	26 (74.3%)	26 (74.3%)
	Inconclusive results	3 (8.6%)	2 (5.7%)
	Mucinous carcinoma	0 (0%)	1 (2.9%)
	Mucinous cystadenoma	1 (2.9%)	0 (0%)
	Pancreatic neuroendocrine tumours	0 (0%)	1 (2.9%)

Data are presented as mean ± standard deviation or n (%).

Among the 35 patients in Group A, four patients underwent repeat biopsy. Among these patients, two were ultimately diagnosed with pancreatic cancer on the basis of histology. Interestingly, pancreatic cancer was not initially detected in two patients, but they were later diagnosed with pancreatic cancer on the basis of clinical follow-up. In Group B, one patient was diagnosed with pancreatic cancer on the basis of histology obtained from a repeat biopsy.

The rate of inconclusive pathological results differed between Group A and Group B, although the difference was not statistically significant (p > 0.05). In Group A, 2 patients (5.7%) had inconclusive pathological diagnoses. In Group B, 3 patients (8.6%) had similar findings. Those patients were ultimately diagnosed with pancreatic cancer on the basis of clinical follow-up and the detection of elevated CA19–9 levels.

There was no significant difference in age, sex, location or size of the lesion, hypoechoic appearance, repeat biopsy or the incidence of severe complications between the US-FNA and EUS-FNA groups. In Group A, adequate specimens were obtained from all 35 patients, accounting for a 100% success rate. In contrast, 29 out of 35 patients (82.9%) in Group B had adequate specimens (p < 0.05). This finding indicates a statistically significant difference in specimen acquisition between the two groups.

**Fig. 3:**
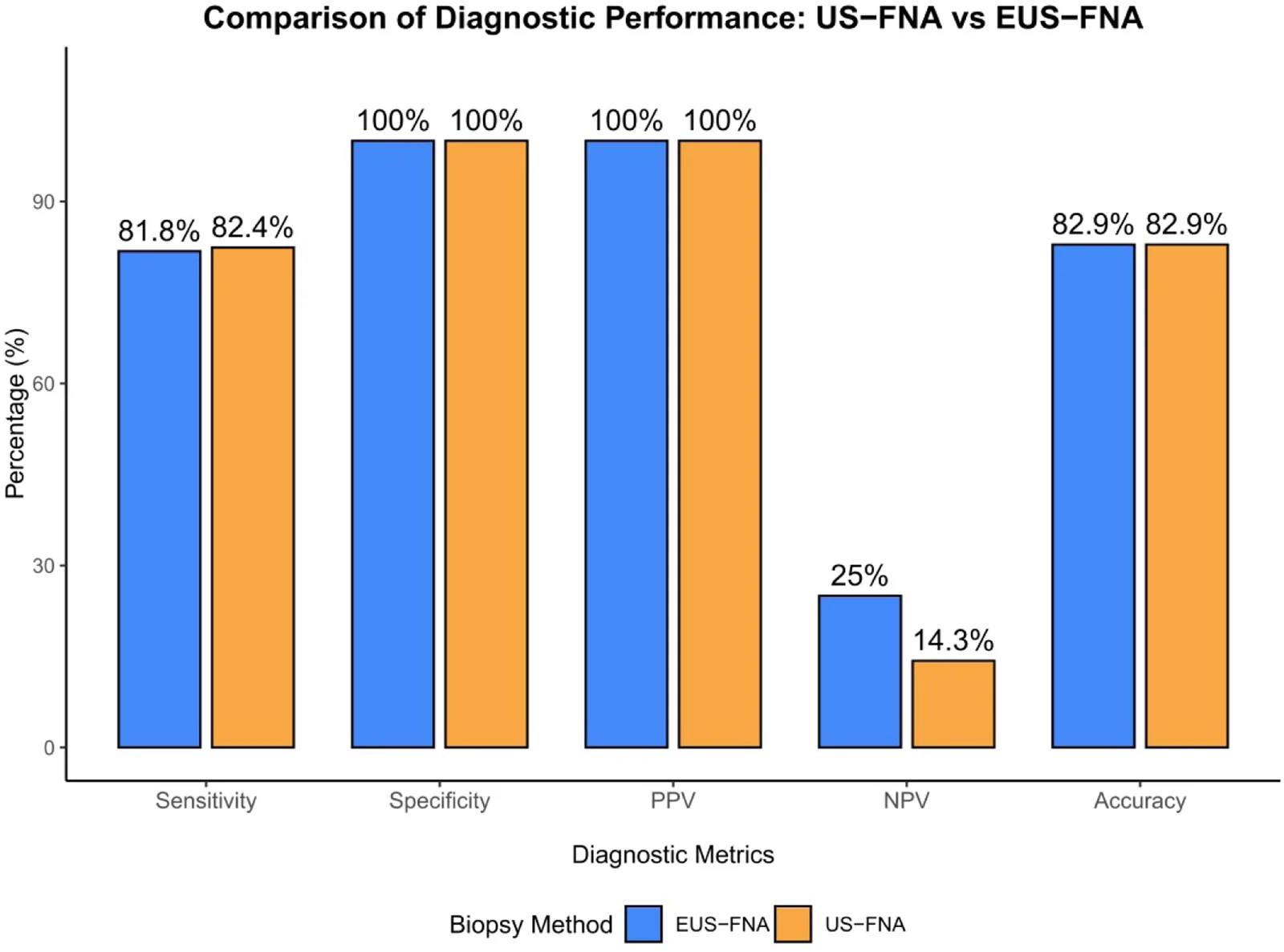
Comparison of diagnostic performance: US-FNA vs. EUS-FNA.

The diagnostic accuracy in Group A was 82.9%, whereas in Group B, it was 82.9%. With respect to sensitivity, specificity, Positive Predictive Value (PPV), and Negative Predictive Value (NPV), the values for Group A are as follows: sensitivity: 82.4%, specificity: 100%, PPV: 100%, NPV: 14.3%. For Group B, the values are as follows: sensitivity: 81.8%, specificity: 100%, PPV: 100%, and NPV: 25%, as shown in Fig. 3.

Complications occurred in 2 (5.8%) patients in Group A and 1 (2.9%) patient in Group B (p > 0.05). In Group A, one patient developed a fever and another patient experienced intra-abdominal infection. These patients recovered with symptomatic treatment. In contrast, one patient in Group B experienced pancreatitis.

## Discussion

For patients who are not surgical candidates, establishing a definitive diagnosis through effective detection methods is important,^7^ while precise diagnosis now hinges on profiling key biomarkers ‒ including KRAS, MSI, and PD-L1^8^ ‒ alongside traditional morphology. Given the progress in modern oncology, targeted therapy and immunotherapy have emerged as promising treatment modalities. Consequently, the acquisition of adequate tissue samples for comprehensive genetic testing and molecular profiling is becoming major method for treatment.^9^

However, obtaining samples from the pancreas may be challenging. Both US-FNA and EUS-FNA are commonly employed modalities for diagnosing pancreatic malignancies. Horwhat et al. conducted a randomized comparative study to evaluate the diagnostic accuracy of EUS-FNA versus CT/US-FNA and reported that EUS-FNA had an accuracy of 88.9% versus 72.2% for CT/US-FNA.^10^ Additionally, some retrospective studies have suggested that EUS-FNA has greater accuracy than US-FNA does.^11^^,^^12^ However, the diagnostic accuracy has been reported to be 89.8% in the EUS-FNA group and 95.2% in the US-CNB/FNA group for the diagnosis of pancreatic malignancies.^13^

In our centre, the operator performed US-FNA or EUS-FNA on the basis of the size and location of the lesion. A 22-gauge cutting needle was used for EUS-FNA, and an 18-gauge needle was used for US-FNA. In our research, adequate specimens were obtained from 35 US-FNA patients (100%) and 29 EUS-FNA patients (82.9%) (p < 0.05). While it is possible for larger-gauge needles to obtain larger and more intact tissue samples in theory, the operation is often more challenging for operator. Several studies have found no significant difference in performance between needle gauges.^14^ Sufficient sample volume from US-FNA is advantageous for supporting molecular or genetic analyses. Nonetheless, evidence confirms that both US-FNA and EUS-FNA can provide adequate material for molecular and genomic profiling.^15^^,^^16^ Physicians often believe that EUS-FNA is the preferred modality when the average lesion size is <30 mm.^17^, ^18^, ^19^ Due to the limited sample size of only 19 cases with lesions smaller than 30 mm, this study was underpowered to detect potentially meaningful differences between the two methods. The future of cancer biopsy lies in integrating diagnostic accuracy with the ability to support molecular profiling.^20^ Our findings highlight that the selection of biopsy technique (US-FNA vs. EUS-FNA) should not only be based on diagnostic performance but also on the clinical need for downstream molecular testing.

In this study, We administered prophylactic octreotide because our patients carried high-risk factors for pancreatitis, consistent with evidence from European Society of Gastrointestinal Endoscopy (ESGE)^21^^,^^22^ recommending this approach to mitigate serious complications. Agkistrodon serum which is part of our routine protocol facilitates thrombin generation, and domestic studies confirm its effectiveness in reducing surgical bleeding.^23^ A meta-analysis supports NSAIDs for preventing pancreatitis after pancreatic procedures.^24^ Additionally, multiple multicenter randomized controlled trials demonstrate that proton-pump inhibitors such as omeprazole lower the incidence of stress ulcers and bleeding.^25^ Accordingly, we employed these medications in our study to reduce procedure-related complications. we also assessed the complication rates associated with US-FNA and EUS-FNA procedures (5.8%vs. 2.9%). The reported complications included pancreatitis, fever and intra-abdominal infection. There were no cases of cancer seeding in the US-FNA or EUS-FNA groups. Few cases of needle tract seeding were reported in either group.^26^, ^27^, ^28^

Further research and clinical experience are necessary to fully evaluate the diagnostic yield and efficacy of US-FNA in the context of pancreatic cancer diagnosis and management. However, there are several limitations in our study. First, our research is a retrospective study. Secondly, we apply propensity score matching to control for selection bias. However, we acknowledge that the generalizability of our conclusions is constrained by the trial's single-center design and relatively small number of participants. Lastly, this study was not designed to evaluate pathological outcomes from different procedural approaches. Defining how biopsy method selection influences the success of subsequent molecular and genetic analyses remains an essential goal for future research, given its growing importance in the management of pancreatic cancer.

## Conclusion

In conclusion, this study provides valuable insights into the comparative effectiveness and safety of EUS-FNA and US-FNA in the diagnosis of pancreatic malignancy. Both techniques demonstrated a high diagnostic yield, with US-FNA proving to be an effective and safe option in comparison with EUS-FNA. Further research and larger studies are warranted to validate these findings and provide more comprehensive evidence for the selection of the most appropriate biopsy technique in the diagnosis of pancreatic malignancy.

## Ethics approval and consent to participate

Ethical approval (20260709-010) was granted by the First Affiliated Hospital of Chongqing Medical University (Chongqing, China).

## Authors’ contributions

Shiming Jiang: Conceptualization; Methodology; Software.

Zhaowei Wu.: Data curation; Writing-original draft preparation.

Panguo Wang: Visualization; Investigation.

Yong Chen: Supervision.

Yachao Zhu: Writing-reviewing and editing.

## Funding

This research did not receive any specific grant from funding agencies in the public, commercial, or not-for-profit sectors.

## Conflicts of interest

The authors declare no conflicts of interest.

## Data Availability

The data must be requested from the corresponding author.
